# Design, method and application of stopping rules in a phase III 2x2 factorial randomised controlled trial

**DOI:** 10.1186/1745-6215-16-S2-P207

**Published:** 2015-11-16

**Authors:** Megan Bowers, Louise Stanton, Mark Thursz

**Affiliations:** 1 Southampton Clinical Trials Unit, University of Southampton, Southampton, UK; 2 Imperial College, London, UK

## Background

The STOPAH trial was a double-blind, 2x2 factorial, phase III randomised clinical trial assessing the treatment of prednisolone and pentoxifylline in patients with severe alcoholic hepatitis; a study that aimed to evaluate 28-day mortality [1].

At the time of trial development, the benefits/harms of pentoxifylline in this patient population were unknown. Prednisolone had a significant though uncertain evidence base. No treatment interaction was assumed yet there was uncertainty about its effect. Therefore, pre-specified stopping rules to assist decision making in stopping treatment arms were required.

## Methods

There was no published methodology on stopping rules for factorial trials, so a design based on the Peto-Haybittle [2] rule was created. Logistic regression modelling 28-day mortality, adjusting for factorial design, was to be produced at interim time points. Treatment arms were to be stopped if the two-sided p-value was <0.001. Both benefit and harm (symmetrical stopping boundary) treatment effects were evaluated. This conservative method was formulated because the treatments were already in practice and so convincing evidence to stop early was needed. The treatment interaction of prednisolone and pentixoifylline was assessed at the 10% significance level. If significant, the factorial design assumption was to be removed prior to interim analysis. The stopping rules would then be applied as described.

## Application

An independent data monitoring committee assessed these stopping rules after 200, 400 and 800 patients had reached the primary endpoint. The stopping rules were not met at any of the three interim looks. The results of the STOPAH trial have recently been published [3].

## References

[B1] Forrest Steroids or pentoxifylline for alcoholic hepatitis (STOPAH): study protocol for a randomised controlled trial Trials 2013 14 262 23958271 10.1186/1745-6215-14-262 PMC3766225

[B2] Pocock SJ Current controversies in data monitoring for clinical trials Clin Trials 2006 3 513 17170035 10.1177/1740774506073467

[B3] Thursz Prednisolone or Pentoxifylline for Alcoholic Hepatitis N Engl J Med 2015 372 1619 1628 25901427 10.1056/NEJMoa1412278

